# Polymorphisms in the Angiogenesis-Related Genes *EFNB2*, *MMP2* and *JAG1* Are Associated with Survival of Colorectal Cancer Patients

**DOI:** 10.3390/ijms21155395

**Published:** 2020-07-29

**Authors:** Dominique Scherer, Heike Deutelmoser, Yesilda Balavarca, Reka Toth, Nina Habermann, Katharina Buck, Elisabeth Johanna Kap, Akke Botma, Petra Seibold, Lina Jansen, Justo Lorenzo Bermejo, Korbinian Weigl, Axel Benner, Michael Hoffmeister, Alexis Ulrich, Hermann Brenner, Barbara Burwinkel, Jenny Chang-Claude, Cornelia M. Ulrich

**Affiliations:** 1 Division of Preventive Oncology, National Center for Tumor Diseases (NCT) and German Cancer Research Center (DKFZ), 69117 Heidelberg, Germany; scherer@imbi.uni-heidelberg.de (D.S.); heike.deutelmoser@nct-heidelberg.de (H.D.); ybalavarca@gmail.com (Y.B.); r.toth@dkfz-heidelberg.de (R.T.); nina.habermann@embl.de (N.H.); katharina.buck@gmx.de (K.B.); akkebotma@hotmail.com (A.B.); h.brenner@dkfz-heidelberg.de (H.B.); 2 Institute of Medical Biometry and Informatics, University of Heidelberg, 69117 Heidelberg, Germany; lorenzo@imbi.uni-heidelberg.de; 3 Division of Cancer Epigenomics and Cancer Risk Factors, German Cancer Research Center (DKFZ), 69117 Heidelberg, Germany; 4 European Molecular Biology Laboratory (EMBL), Genome Biology, 69117 Heidelberg, Germany; 5 Division of Cancer Epidemiology, German Cancer Research Center (DKFZ), 69117 Heidelberg, Germany; lisannekap@gmail.com (E.J.K.); p.seibold@Dkfz-Heidelberg.de (P.S.); j.chang-claude@dkfz-heidelberg.de (J.C.-C.); 6 Division of Clinical Epidemiology and Aging Research, German Cancer Research Center (DKFZ), 69117 Heidelberg, Germany; l.jansen@Dkfz-Heidelberg.de (L.J.); k.weigl@dkfz-heidelberg.de (K.W.); m.hoffmeister@dkfz-heidelberg.de (M.H.); 7 Division of Biostatistics, German Cancer Research Center (DKFZ), 69117 Heidelberg, Germany; benner@dkfz-heidelberg.de; 8 Department of General, Visceral and Transplantation Surgery, University Hospital Heidelberg, 69117 Heidelberg, Germany; aulrich@lukasneuss.de; 9 Chirurgische Klinik I, Lukaskrankenhaus Neuss, 41464 Neuss, Germany; 10 German Cancer Consortium (DKTK), German Cancer Research Center (DKFZ), 69117 Heidelberg, Germany; 11 Division of Molecular Epidemiology, German Cancer Research Center (DKFZ), 69117 Heidelberg, Germany; B.Burwinkel@dkfz-heidelberg.de; 12 Division Molecular Biology of Breast Cancer, Department of Gynecology and Obstetrics, University of Heidelberg, 69117 Heidelberg, Germany; 13 Cancer Epidemiology Group, University Cancer Center Hamburg, University Medical Center Hamburg-Eppendorf, 20246 Hamburg, Germany; 14 Huntsman Cancer Institute, Salt Lake City, UT 84112, USA; 15 Department of Population Health Sciences, University of Utah, Salt Lake City, UT 84112, USA

**Keywords:** angiogenesis, colorectal cancer, survival, single nucleotide polymorphism

## Abstract

An individual’s inherited genetic variation may contribute to the ‘angiogenic switch’, which is essential for blood supply and tumor growth of microscopic and macroscopic tumors. Polymorphisms in angiogenesis-related genes potentially predispose to colorectal cancer (CRC) or affect the survival of CRC patients. We investigated the association of 392 single nucleotide polymorphisms (SNPs) in 33 angiogenesis-related genes with CRC risk and survival of CRC patients in 1754 CRC cases and 1781 healthy controls within DACHS (Darmkrebs: Chancen der Verhütung durch Screening), a German population-based case-control study. Odds ratios and 95% confidence intervals (CI) were estimated from unconditional logistic regression to test for genetic associations with CRC risk. The Cox proportional hazard model was used to estimate hazard ratios (HR) and 95% CIs for survival. Multiple testing was adjusted for by a false discovery rate. No variant was associated with CRC risk. Variants in *EFNB2*, *MMP2* and *JAG1* were significantly associated with overall survival. The association of the *EFNB2* tagging SNP rs9520090 (*p* < 0.0001) was confirmed in two validation datasets (*p*-values: 0.01 and 0.05). The associations of the tagging SNPs rs6040062 in *JAG1* (*p*-value 0.0003) and rs2241145 in *MMP2* (*p*-value 0.0005) showed the same direction of association with overall survival in the first and second validation sets, respectively, although they did not reach significance (*p*-values: 0.09 and 0.25, respectively). *EFNB2*, *MMP2* and *JAG1* are known for their functional role in angiogenesis and the present study points to novel evidence for the impact of angiogenesis-related genetic variants on the CRC outcome.

## 1. Introduction

Colorectal cancer (CRC) is the second most common cancer and the second leading cause of cancer death among men and women throughout the world asserting major public health problems [1]. Disease risk and prognosis are to a large proportion modifiable with obesity, red meat consumption and sedentary lifestyle increasing the risk and physical activity, NSAID (non-steroidal anti-inflammatory drug) use and fiber intake being protective [2,3,4]. However, there is also a genetic component to the disease, which is estimated to explain up to 35% of the heritability in colorectal cancer risk [5,6]. Some studies point to genetic associations with the CRC outcome [7,8,9]. However, evidence is still limited.

Angiogenesis, the generation of new blood vessels from pre-existing vessels, is crucial for tumor growth, progression and metastasis as it provides nutrients and oxygen to the growing tumor [10]. While in normal tissue, angiogenesis is tightly controlled and only transiently turned on, in carcinogenic progression an ‘angiogenic switch’ perpetuates the formation of new blood vessels [11].

VEGF (vascular endothelial growth factor) as one of the most potent endothelial cell mitogens is a major driver of angiogenesis. It can act in an autocrine or paracrine manner through interaction with its receptors (VEGFR1-3, also named FLT1 (fms related tyrosine kinase 1), KDR (kinase insert domain receptor) and FLT4 (fms related tyrosine kinase 4), respectively) and co-receptors (neuropilins, NRPs) [12]. Such interactions may further be regulated through ephrinB2 (EFNB2) and finally stimulate proliferation, migration of endothelial cells, cell degradation, remodeling of the extracellular matrix (ECM) and angiogenesis [13]. Ephrins and their receptors (Eph receptors) are crucial in numerous biological processes [14]. Their bidirectional signaling triggers cascades in both, the receptor- and the ligand-bearing cells and deregulated Eph/ephrin activation as well as altered expression of the receptors or their ligands have been frequently reported in a variety of tumors including colorectal neoplasms [14,15]. Based on these observations, targeted interference with Eph/ephrin signaling has been proposed for cancer therapy [16].

Matrix metalloproteinases (MMPs) are critical in ECM remodeling [13,17]. In addition to their capability to promote bioavailability of VEGF, the proteolytic activities of MMPs facilitate protein degradation and support vessel formation [18]. VEGF signaling also regulates DLL4 expression, which is one of two key ligands of NOTCH4 (notch receptor 4), the second ligand being JAG1 (jagged canonical Notch ligand 1) [19]. Concurrently, NOTCH4 promotes VEGFR expression [20].

An individual’s inherited genetic background may modify angiogenesis-related signaling cascades that affect blood supply of premalignant lesions and/or macroscopic tumors [21]. We hypothesize that this may result in altered cancer risk or disease outcome after colorectal cancer diagnosis.

In the present study, we investigated the association between angiogenesis-related genetic variants and risk of CRC, overall survival (OS) and recurrence-free survival (RFS) of colorectal cancer patients in 1754 CRC cases and 1781 healthy controls using a comprehensive targeted genotyping approach and validated some of the associations.

## 2. Results

In this study 392 SNPs in 33 angiogenesis-related genes (Table S1) were investigated for their association with risk of colorectal cancer and survival of colorectal cancer patients in a population of 1754 colorectal cancer cases and 1781 healthy individuals (Tables S2, S3 and S4, respectively). Characteristics of the study population are presented in Table 1.

### 2.1. Risk

Higher education and the use of NSAIDs were associated with reduced risk of colorectal cancer. In addition, cases were more likely to smoke regularly, consume higher amounts of red meat and alcohol and were more often diabetic and obese (Table 1a).

No variant was associated with the risk of colorectal cancer after adjustment for multiple testing (Table S2). Furthermore, we did not observe statistically significant interactions of any of the investigated polymorphisms with NSAID use or smoking in relation to the risk of colorectal cancer.

### 2.2. Overall and Recurrence-Free Survival

During a median follow up of five years, 538 patients died and 468 had a recurrent event. Deceased patients were older, diagnosed with higher disease stages and tumor grade. They were less likely to consume alcohol and to be overweight or obese (Table 1b).

Eleven tagging SNPs in four genes (*EFNB2*, *MMP2*, *JAG1* and *KDR*) were significantly associated with overall survival (Table 2, Figure S1 and Table S3) of colorectal cancer patients. Five variants in *EFNB2*, none of which were in high LD (r^2^ < 0.8), were associated with overall survival. The strongest associations with overall survival were observed for the protective variant rs2391333 (HR_C > T_: 0.77, 95% CI: 0.68–0.87, *p*-value: <0.0001) and for the risk variant rs9520090 (HR_G > C_: 1.29, 95% CI: 1.15–1.46, *p*-value: <0.0001). Furthermore, rs9520087 (HR_G > A_: 1.24, 95% CI: 1.10–1.40, *p*-value: 0.0006), rs9520088 (HR_TT_ vs. _TC/CC_: 1.37, 95% CI: 1.15–1.62, *p*-value: 0.0003) and rs2057408 (HR_AA_ vs. _AG/GG_: 1.35, 95% CI: 1.14–1.62, *p*-value: 0.0008) were significantly associated with worse overall survival (Table 2a). All associations with overall survival remained statistically significant after correction for multiple testing (*q* < 0.05).

Four tagging SNPs (none in high LD with r^2^ < 0.8) in the region of *MMP2* were significantly associated with overall survival of colorectal cancer patients (Table 2a). Rs17301608 was significantly associated with poorer OS (HR_A > G_: 1.25, 95% CI: 1.11–1.41, *p*-value: 0.0003, Table 2a). The variants rs2241145 (HR_G > C_: 1.23, 95% CI: 1.09–1.38, *p*-value: 0.0005) and rs1561219 (HR_C > A_: 1.32, 95% CI: 1.11–1.56, *p*-value: 0.0009) were also associated with decreased overall survival. In contrast, the variant rs243847 significantly improved overall survival (HR_T > C_: 0.81, 95% CI: 0.71–0.92, *p*-value: 0.0010). All associations remained statistically significant after multiple testing correction (*q* < 0.05).

One intronic variant in *JAG1* (rs6040062) was significantly associated with shorter overall survival (HR_G > C_: 1.36, 95% CI: 1.15–1.61, *p*-value: 0.0003, Table 2a) and recurrence-free survival (HR_G > C_: 1.31, 95% CI: 1.09–1.57, *p*-value: 0.004, Table S4) of colorectal cancer patients. Only the association with overall survival remained significant after multiple testing correction (*q* < 0.05).

Finally, the V297I variant (rs2305948) in *KDR* was associated with decreased overall survival (HR_CC_ vs. _CT/TT_: 1.28, 95% CI: 1.04–1.57, *p*-value: 0.02, Table 2b). For its deleterious and probably damaging SIFT and PolyPhen scores, respectively, this polymorphism was a selected candidate.

SNPs in *MMP2*, *EFNB2* and *JAG1,* which were significantly associated with patients’ survival, were subsequently analyzed in a first validation set of 2165 (for *MMP2* and *JAG1*), and 1638 (for *EFNB2*) DACHS colorectal cancer patients and a second validations set of 372 colorectal cancer patients from TCGA where genome-wide data was available.

In the first validation set of imputed genotypes from the DACHS study with a median follow up of five years, the association with rs9520090 in *EFNB2* was validated in a dominant model (HR_GG_ vs. _GC/CC_: 1.22, 95% CI: 1.00–1.47, *p*-value: 0.05). The variant rs6040062 in *JAG1* showed a similar direction (HR: 1.10) in the validation set, however the association was not significant (*p*-value: 0.25). No other association was validated in this dataset (Table S5).

For the second validation set, we retrieved data of 372 colorectal cancer patients with a median follow up of 0.6 years from The Cancer Genome Atlas (TCGA) Colon Adenocarcinoma (COAD) and Rectal Adenocarcinoma (READ) to validate significant associations in an external dataset. The variant rs7983579, which is in LD with rs9520090 (r^2^ = 0.8) in *EFNB2,* was significantly associated with the overall survival of colorectal cancer patients (HR_T > G_: 1.76, 95% CI: 1.14–2.73, *p*-value: 0.01). The variant rs9520090 itself showed the same direction of association with overall survival, although it did not reach significance (HR_G > C_: 1.28, 95% CI: 0.84–1.94, *p*-value: 0.25). Furthermore, the variants rs9923304 (HR_T > C_: 1.49, 95% CI: 0.94–2.38, *p*-value: 0.09) and rs1477017 (HR_A > G_: 1.42, 95% CI: 0.91–2.22, *p*-value: 0.12) in *MMP2* linked to rs2241145 (r^2^ = 0.83) and rs17301608 (r^2^ = 0.93), respectively, were marginally associated with overall survival. No TCGA data were available for variants in *JAG1*.

The gene-environment interaction between overall survival and smoking, NSAID use, adjuvant 5-FU-based chemotherapy and microsatellite status was investigated for all variants (Table S7). Some indication for interaction was observed for two tagging SNPs in *ETS1* (rs11221322 and rs7121854: *p*_interaction_: 0.0004 and 0.001, respectively) and the candidate missense variant in *TEK* V486I (*p*_interaction_: 0.003) with MSI (microsatellite instability), for the candidate missense variant *KDR* Q472H (rs1870377; *p*_interaction_: 0.03) with smoking, for the candidate missense variant *TEK* V486I (rs1334811, *p*_interaction_: 0.03) with NSAID use and for the *IL10* candidate rs1800890 (*p*_interaction_: 0.01) with adjuvant 5-FU-based chemotherapy for overall survival of colorectal cancer patients. These associations did not remain significant after adjustment for multiple testing.

## 3. Discussion

Genetic variation may contribute to an ‘angiogenic switch’, which is essential for blood supply and tumor growth in microscopic and macroscopic tumors. In the present study, we investigated the association of polymorphisms in angiogenesis-related genes with the risk of colorectal cancer as well as with overall survival and recurrence-free survival of colorectal cancer patients in the DACHS population-based case-control study using 1754 CRC cases and 1781 controls. We chose a targeted candidate gene approach to obtain a more complete coverage of the hypothesized genes and avoided potentially high numbers of false-negative findings, which are often the result of large-scale genotyping studies. We observed significant associations between variants in *EFNB2*, *MMP2* and *JAG1* and overall survival. Some of the associations could be validated.

Ephrins are membrane-associated ligands that function through interaction with their receptors, erythropoietin-producing hepatocellular (Eph) receptor tyrosine kinase. The unique ligand-receptor interaction, which requires cell-cell contact, can trigger bidirectional signaling, with effects in both the ligand- and the receptor-bearing cell [22]. Downstream or upstream signaling cascades regulate cell proliferation, migration, adhesion, differentiation, survival and angiogenesis and are crucial for homeostasis in epithelial tissues. In this study, we observed significant associations between five variants in ephrin B2 (*EFNB2*) and OS of colorectal cancer patients. Rs9520087 is located in the 3′UTR of *EFNB2*, which may suggest regulatory effects on gene expression of *EFNB2*, however, the in silico functional follow up did not reveal any associations with gene expression. It is not linked to any known variant and it is covered only by a small number of genome-wide genotyping arrays. Imputation of the variant in a validation set of 2165 CRC cases resulted in 10% of inaccurately imputed alleles, which may be the reason why this variant has not been identified earlier [23]. Similarly, other *EFNB2* variants are only present on a limited number of genome-wide genotyping arrays. Rs3742159, linked to rs9520088 (r^2^ = 1) displayed a RegulomeDB rank of 3a. It is located within a region, which likely affects binding of transcription factors, one of which was identified to be NFKB [24]. None of the other linked SNPs seems to be functionally important. The SNPs rs2391333 and rs9520090 modify the methylation status of the site chr13:107163855 (methylation probe cg05493945) within *EFNB2*. Whether this results in changed gene expression of *EFNB2* needs to be further investigated. A number of studies have shown that *EFNB2* and its receptor *EPHB4* are transcriptionally upregulated in colon cancer carcinoma cell lines and in colon cancer tissue as compared to adjacent mucosa, supporting a role of EFNB2 in carcinogenesis potentially through modulation of angiogenic signals, even though the exact mechanisms remain to be fully elucidated [25]. No other gene, which may be relevant for cancer progression is located in the vicinity of this region, thus it seems plausible, that the identified associations with survival of colorectal cancer patients are linked to *EFNB2*.

We observed a statistically significant association with OS of colorectal cancer patients for four *MMP2* tagging SNPs (rs17301608, rs2241145, rs1561217 and rs243847), all located in introns or in non-coding exon regions, while a previous association of the *MMP2* rs243865 (−1306 C > T-in our study the linked SNP rs243866 was genotyped, r^2^: 0.99) in smaller studies of OS or CRC risk was not confirmed [26,27]. The major task of MMPs is the degradation of proteins, which is an essential step in many hallmarks of cancer in regulating cell growth, differentiation, apoptosis, migration, invasion, immune surveillance and cancer angiogenesis [18]. Consequently, MMPs play a crucial role in malignant transformation and cancer progression. To our knowledge, neither of the polymorphisms or any of the linked variants has previously been reported for their association with OS of colorectal cancer patients. In silico functional follow up of rs17301608 revealed that three of the linked variants (rs1477017, rs11646643 and rs9302671; r^2^ > 0.8) ranked low (1f) at RegulomeDB, supporting a regulatory role. They likely affect the binding of transcription factors (TF), potentially of NR2F2 (nuclear receptor subfamily 2 group F member 2), which was previously shown to regulate cell growth, invasiveness, metastasis and angiogenesis [28,29]. Furthermore, all three variants linked to rs17301608 are reported expression quantitative trait loci (eQTLs) for *LPCAT2* (lysophosphatidylcholine acyltransferase 2), which was previously associated with the progression of breast cancer, ovarian cancer and CRC in human tissue and in cell lines [30]. Finally, data from TCGA supports the function of rs2241145 and rs17301608 as eQTLs on *LPCAT2* expression in prostate adenocarcinoma [31].

Aberrant NOTCH signaling promotes the development and progression of colorectal cancer and is partly driven by the overexpression of JAG1, a ligand of NOTCH4 [32]. We identified one tagging SNP (rs6040062) in *JAG1*, which was associated with shorter OS of colorectal cancer patients. The variant and its linked SNPs rs3748480 (r^2^: 1.0) and rs112946915 (r^2^: 0.97) are intronic. The lowest functional RegulomeDB rank was reached by rs112946915 (3a), which modifies binding motifs for transcription factors STAT1 and IRF1 among others. JAG1 has been considered as an attractive target for colorectal cancer therapy, as it seems to be a more specific target than other members of the NOTCH pathway [33].

Our results are based on more than 1700 CRC cases and 1700 healthy controls, with comprehensive clinical, epidemiological and demographic data, which is a major strength of the study. The detailed information on lifestyle factors as well as the availability of clinical patients’ characteristics enabled us to adequately account for relevant environmental factors and test for effect modification. Although our sample size was fairly large, some of the interaction analyses were based on small strata. Unfortunately, the MSI status was missing for 22% of the population and 9% of investigated tumors were MSI-high. Thus, associations should be considered with caution. Furthermore, we had access to two validation sets and validated the association of the *EFNB2* tagging SNP rs9520090 (or its linked variant rs7983579) with the overall survival of colorectal cancer. In addition, tagging SNPs in *JAG1* and *MMP2* showed the same direction of association with overall survival in the first and second validation set, respectively.

The signals of the first validation set were weaker than we expected considering that the discovery and first validation sets were derived from the same study population. We thus compared both sample sets with respect to genetic, epidemiological and clinical data. For up to 67 individuals, imputed genotypes from genome-wide data and at the same time directly genotyped genotypes were available. We observed a discrepancy of 5%–25% between the directly genotyped and imputed genotypes, suggesting an insufficient imputation accuracy for these regions and supporting the choice of a targeted candidate gene approach. The accuracy of imputed genotypes strongly relies on linkage disequilibrium (LD), consequently they depend on the genotypes measured within the same LD-block. *EFNB2* and *JAG* are located in regions of rather low LD, which reduces the probability to correctly impute missing genotypes [34]. We further observed differences between the two study sample sets with respect to overall survival, alcohol consumption and tumor grade of patients. We adjusted for these variables, however, other confounders not captured within this study may explain the discrepant association strengths between the two study samples. While 98% of participants reported to be of German nationality, the ethnic background of study participants was not assessed as part of this study. We cannot rule out an effect of ethnicity on the identified associations, even though this is likely to be very small, because they are expected to be of Caucasian origin.

In conclusion, we identified novel genetic associations with the overall survival of colorectal cancer patients. To our knowledge, no previous study has reported associations between SNPs in *EFNB2* or *JAG1* with the CRC outcome, both genes known for their functional role in angiogenesis and their impact on disease progression. The present study cannot point to the causal variant or elucidate the functional role of the identified polymorphisms and additional investigations in other populations are required to further validate these associations and to unravel the causal variants and their functionalities.

## 4. Materials and Methods 

### 4.1. Study Population

The study population includes patients with colorectal cancer who participated in a long-term follow-up study of patients of the German population-based case-control study DACHS (“Darmkrebs: Chancen der Verhuetung durch Screening”; described in detail elsewhere) [35,36,37,38]. In brief, colorectal cancer patients with a primary, confirmed diagnosis of colorectal cancer were recruited from hospitals of the Rhein-Neckar-Odenwald region 2003–2007 if they were aged 30 years or older, resident in the study region and able to complete an in-person interview. Baseline interviews were performed using standardized questionnaires to collect information on demographic and established or suggested colorectal cancer risk factors as well as possible prognostic factors. Follow-up information on vital status was collected at three, five and ten years after diagnosis. Causes of death were verified by death certificates and coded based on ICD−10 classifications. Information on recurrences and secondary tumors were collected from general practitioners and specialists as applicable. In addition, clinical and histological data were extracted from patient and pathological records. Patients without baseline blood samples (post-diagnostic) or without mouthwash (<1%) were excluded from the study.

Population controls were randomly selected from lists of residents of the population registries of the cities and counties. Controls were matched to cases by sex, county of residence and 5-year age group. Controls were eligible if they were aged 30 years or older with no history of colorectal cancer.

The study was approved by the ethics committee (approved on 6 December 2001, project identification code 310/2001) of the University of Heidelberg and State Medical Boards of Baden-Wuerttemberg and Rhineland-Palatinate and was conducted in agreement with the Helsinki Declaration. Written informed consent was provided by all participants at the baseline and during follow-up.

### 4.2. Single Nucleotide Polymorphism (SNP) Selection and Genotyping

We selected 33 genes related to angiogenesis based on a comprehensive literature search, gene ontology data bases and external expertise. The genes comprised: *AKT1, ANGPT1, ANGPT2, DKK4, DLL1, DLL4, EFNB2, EPHB4, ETS1, FGF2, FLT1, FLT4, HIF1A, ID1, JAG1, KDR, MAPK1, MMP2, MMP9, NOTCH4, NRP1, NRP2, PDGFA, PDGFRB, PGF, PIK3CA, PIK3CG, TEK, TIE1, VEGFA, VEGFB, VEGFC* and *VHL* (Table S1).

We used Haploview 4.2 (Broad Institute, Cambridge, MA, USA) to identify tagging SNPs located in the assigned genes as well as in the 5′ and 3′ flanking regions (up to 10 kb) for coverage of the genetic variation across the gene. The tagging SNP selection was based on the HapMap project (CEU [Utah Residents (CEPH) with Northern and Western European Ancestry] population, Phase II/Release 24), using a pairwise tagging approach applying r^2^ ≥ 0.8 as the cutoff.

Genomic DNA was extracted from EDTA (Ethylenediaminetetraacetic acid) anticoagulated blood or mouthwash samples using the FlexiGene DNA kit (Qiagen GmbH, Hilden, Germany) and quantified using Quant-iT PicoGreen dsDNA reagent and kit (Invitrogen/Life Technologies, Darmstadt, Germany). Genotyping was performed using the Illumina GoldenGate assay or iPLEX assay (Sequenom, Hamburg, Germany) for the MassArray system. For quality control, all assays were validated using the 72 CEPH controls, distributed randomly among the study samples. Each assay batch contained negative and positive controls and 5% of the total number of samples were re-genotyped to confirm reproducibility. Call rates for all genotypes exceeded 96%. The concordance for blinded duplicates was >99.7%. Deviation from the Hardy-Weinberg equilibrium was evaluated using a χ2 statistic in all control subjects. A total of 392 single nucleotide polymorphisms in 33 genes were investigated in this study, which passed quality control, were not in linkage disequilibrium (LD) and had a cell count of at least 10.

### 4.3. Imputation

Imputation of environmental factors was performed using multiple imputation as most variables, except for tumor grade (11%), had only few missing values (<1%). For grading, we compared no imputation and multiple imputation of grading with best-case (all missing values set to grade 1) and worst-case (all missing values set to grade 4) resulting in similar effect estimates (<10% difference). Genotype imputation in the DACHS study was performed by using IMPUTE2 and the 1000 Genomes reference panel [39,40].

### 4.4. Statistical Analysis

The associations between polymorphisms and the risk of colorectal cancer were assessed using unconditional logistic regression models estimating odds ratios (OR) and 95% confidence intervals (CI). Associations were also adjusted by relevant environmental factors, which were obtained using the backward elimination procedure. These factors were: age (<60, 60–69, 70–79 and 80+ years), sex, alcohol consumption (no alcohol consumption, 0.1–5.7, 5.7–13.3, 13.3–28.6 and ≥28.6 g alcohol/day during lifetime), BMI category 5–14 years ago (≤18.5 kg/m^2^, 18.5 to < 25kg/m^2^ (reference category), 25 to <30 kg/m^2^ and ≥30 kg/m^2^), first degree family history of CRC (yes, no/unknown), education (low, medium and high), prior colonoscopy (ever colorectal endoscopy after consideration of endoscopy report, yes, no/unknown), diabetes, regular smoking (never, yes), NSAID use (never, ever used NSAIDs more than once/month ≥1 year) and red meat consumption (low, medium and high). 

For each polymorphism, the risk of colorectal cancer was estimated using log-additive, and dominant models. Differences in baseline characteristics between deceased and non-deceased patients were tested using the χ2 statistic and *t*-tests for categorical and continuous variables, respectively. As an outcome, time to survival and recurrence-free survival times were calculated as the time from the diagnosis to the event of interest (death and recurrence or death, respectively) or censoring. Median follow-up time was calculated using the reverse Kaplan-Meier estimator and the cumulative survival curves for overall survival were generated using the Kaplan-Meier curves [41].

Cox proportional hazard models were used to estimate hazard ratios (HR) for overall survival and recurrence-free survival, and their 95% CIs associated with genetic variants. Dominant and log-additive modes of inheritance were assumed for the genetic variants. The models also included adjustment for relevant environmental and clinical factors obtained from a backward elimination procedure. The analyses were adjusted for age (<60, 60–70, 70–80 and 80+ years), sex, stage (I-IV), tumor grade (1 and 2 versus 3 and 4), current BMI (<18.5 kg/m^2^, 18.5 to <25 kg/m^2^ (reference category), 25 to <30 kg/m^2^ and ≥30 kg/m^2^), and alcohol intake (no alcohol intake and quartiles: 0.1–6.1, 6.1–15.6, 15.6–32.6 and ≥32.6 g alcohol/day in the last year).

The effect modification was tested by using multiplicative interaction terms and stratified analyses for adjuvant 5-FU-based chemotherapy (yes/no) and microsatellite status (high/low; only for overall survival), NSAID use (never, ever used NSAIDs more than once/month ≥1 year), and smoking (never/yes).

All statistical analyses were two-sided with a significance level of 0.05 and were performed using SAS (version 9.3, SAS Institute, Cary, NC, USA) and R (version 3.0.2, R Foundation for Statistical Computing, Vienna, Austria).

The false discovery rate (FDR, indicated as the *q*-value) correction method was used to adjust for multiple testing of non-candidate polymorphisms [42].

In silico functional follow up of the SNPs (Table S6) that were statistically significantly associated with risk of CRC or overall survival of CRC patients was performed using Haploreg, LDlink, RegulomeDB, PancanQTL, Pancan-meQTL, GTExPortal and (variant effect predictor) VEP [24,31,43,44,45,46,47].

### 4.5. Validation Sets

Validation of significant associations was performed using two datasets. The first set included DNA samples from an independent sample of 2165 DACHS colorectal cancer patients recruited 2007–2010, which were genotyped using Illumina’s Global Screening Array, OmniExpress BeadChip and OncoArray as described previously [48]. Genotypes were imputed separately for each gene (±1.5 MB) and for each array using the Michigan Imputation Server [49]. The Haplotype Reference Consortium (HRC) reference panel (HRC r1.1 2016) has been used for imputation and Eagle for phasing. Subsequently, the imputed genotypes of each array were combined. The second set included genotype data from The Cancer Genome Atlas (TCGA) Colon Adenocarcinoma (COAD) cohort and Rectal Adenocarcinoma (TCGA-READ) cohort, which were downloaded from the NCI Genomic Data Commons.

A statistical analysis from our respective findings was performed for both validation datasets as described above. TCGA analyses were adjusted for the available variables age, sex and stage.

The data that support the findings of this study are available from the corresponding author upon reasonable request.

## Figures and Tables

**Table:** (**a**)

		Controls	Cases	*p*-Value
**Gender (Male/Female)**		1066/715	1027/727	0.43
**Age (Mean ± SD)**		68.72 ± 10.18	68.25 ± 10.42	0.32
**First Degree Relatives With CRC**	*n* (available)	1781	1754	0.009
	Yes (%)	11.3	14.3	
	No (%)	88.7	85.8	
**Education**	*n* (available)	1781	1754	<0.0001
	Low (%)	53.3	62.9	
	Medium (%)	25.3	20.5	
	High (%)	21.4	16.6	
**Red meat Consumption**	*n* (available)	1781	1754	<0.0001
	Low (%)	10.7	8.2	
	Medium (%)	85.1	84.7	
	High (%)	4.2	7.1	
**Alcohol Consumption (g/day)**	*n* (available)	1781	1754	0.012
	no intake	14.7	16.3	
	0.1–5.7	23.4	21.0	
	5.7–13.3	21.1	20.8	
	13.3–28.6	23.6	21.1	
	≥28.6	17.2	20.9	
**Regular Smoking**	*n* (available)	1781	1754	0.011
	Never (%)	52.2	47.9	
	Current (%)	47.8	52.1	
**Diabetes**	*n* (available)	1781	1754	0.0003
	Yes (%)	13.7	18.2	
	No (%)	86.4	81.8	
**BMI (kg/m^2^)**	*n* (available)	1781	1754	<0.0001
	<18.5 (%)	0.7	0.97	
	18.5–25 (%)	37.1	31.07	
	25–30 (%)	47.0	47.32	
	>30 (%)	15.2	20.64	
**NSAID Use**	*n* (available)	1781	1754	<0.0001
	No (%)	63.1	74.0	
	Yes (%)	37.0	26.0	
**Ever Colonoscopy**	*n* (available)	1781	1754	<0.0001
	No (%)	46.9	79.2	
	Yes (%)	53.1	20.8	

**Table:** (**b**)

		Overall Survival (Discovery)		Overall Survival (Validation)	
**Overall *n*/Events**		1675/538		2165/689	
		HR (95% CI)	*p*	HR (95% CI)	*p*
**Gender**	Male	[1]		[1]	
	Female	0.89 (0.75–1.05)	0.16	1.27 (1.09–1.49)	0.003
**Age Category**	<60	[1]		[1]	
	60–69	1.24 (0.94–1.62)	0.13	1.31 (1.00–1.72)	0.047
	70–79	1.45 (1.10–1.90)	0.007	1.84 (1.43–2.36)	<0.0001
	>80	2.84 (2.14–3.77)	<0.0001	3.40 (2.63–4.39)	<0.0001
**Stage at Diagnosis**	I	[1]		[1]	
	II	1.53 (1.10–2.12)	0.01	1.81 (1.38–2.37)	<0.0001
	III	2.73 (2.01–3.69)	<0.0001	2.70 (2.08–3.52)	<0.0001
	IV	13.28 (9.81–17.98)	<0.0001	13.33 (10.26–17.31)	<0.0001
**Grade**	G1-G2	[1]		[1]	
	G3-G4	1.27 (1.06–1.52)	0.01	1.85 (1.57–2.18)	<0.0001
**Alcohol Consumption** **(g/day)**	no intake	[1]		[1]	
	0.1–6.1	0.69 (0.53–0.88)	0.003	0.79 (0.64–0.98)	0.03
	6.1–15.6	0.70 (0.54–0.91)	0.007	0.76 (0.61–0.95)	0.02
	15.6–32.6	0.67 (0.52–0.86)	0.002	0.88 (0.70–1.09)	0.24
	≥32.6	0.74 (0.58–0.96)	0.02	0.93 (0.73–1.18)	0.54
**BMI (kg/m^2^)**	18.5–25>	[1]		[1]	
	<18.5	1.72 (1.075–2.75)	0.02	1.18 (0.71–1.95)	0.53
	25–30>	0.77 (0.64–0.93)	0.007	0.76 (0.65–0.90)	0.002
	≥30	0.71 (0.56–0.91)	0.007	0.78 (0.63–0.96)	0.02

Abbreviations: HR: hazard ratio; CI: confidence interval; BMI: body mass index.

**Table:** (**a**)

						Overall Survival					Recurrence-Free Survival		
Gene	SNP	Location	Alleles	Alive	Deceased	HR ^1^ (95%-CI)	*p*	*q*	Alive	Deceased	HR ^1^ (95%-CI)	*p*	*q*
*EFNB2*	rs2391333	intronic	CC	455	223	[1]			474	204	[1]		
			CT	526	259	0.77 (0.68–0.87)	<0.0001	0.008	538	247	0.85 (0.74–0.97)	0.02	0.73
			TT	174	64				168	70			
			CT/TT	700	323	0.76 (0.64–0.90)	0.002	0.060	706	317	0.83 (0.69–0.99)	0.05	0.91
	rs9520090	intronic	GG	338	134	[1]			339	133	[1]		
			GC	580	270	1.29 (1.15–1.46)	<0.0001	0.008	586	264	1.20 (1.06–1.37)	0.005	0.68
			CC	237	142				255	124			
			GC/GC	817	412	1.42 (1.16–1.73)	0.0006	0.04	841	388	1.2 (1.03–1.56)	0.03	0.68
	rs9520087	3′UTR	GG	369	148	[1]			371	146	[1]		
			GA	562	297	1.24 (1.10–1.40)	0.0006	0.04	575	284	1.15 (1.00–1.31)	0.05	0.86
			AA	224	101				234	91			
			GA/AA	786	398	1.42 (1.17–1.71)	0.0003	0.03	809	375	1.28 (1.05–1.57)	0.14	0.68
	rs2057408	downstream	AA	509	204	[1]			513	200	[1]		
			AG	488	271	1.19 (1.05–1.34)	0.006	0.13	507	252	1.13 (0.99–1.29)	0.07	0.87
			GG	158	71				160	69			
			AG/GG	646	342	1.35 (1.14–1.62)	0.0008	0.04	667	321	1.26 (1.04–1.52)	0.02	0.68
	rs9520088	intronic	TT	638	269	[1]			641	266	[1]		
			TC	429	239	1.24 (1.08–1.41)	0.002	0.06	446	222	1.15 (0.99–1.33)	0.07	0.87
			CC	88	38				93	33			
			TC/CC	517	277	1.37 (1.15–1.62)	0.0003	0.03	539	255	1.24 (1.03–1.49)	0.02	0.68
*MMP2*	rs17301608	intronic	AA	432	180	[1]			431	181	[1]		
			AG	540	267	1.25 (1.11–1.41)	0.0003	0.03	551	256	1.11 (0.98–1.27)	0.11	0.87
			GG	183	99				198	84			
			AG/GG	723	366	1.42 (1.18–1.71)	0.0002	0.03	749	340	1.23 (1.01–1.49)	0.04	0.91
	rs1561219	intronic	CC	877	378	[1]			877	378	[1]		
			CA	255	160	1.32 (1.11–1.56)	0.0009	0.04	277	138	1.11 (0.92–1.34)	0.26	0.89
			AA	23	8				26	5			
			CA/AA	278	168	1.37 (1.14–1.65)	0.0009	0.04	303	143	1.17 (0.95–1.40)	0.14	0.91
	rs2241145	intronic	GG	341	133	[1]			334	140	[1]		
			GC	547	281	1.23 (1.09–1.38)	0.0005	0.04	566	262	1.11 (0.98–1.26)	0.11	0.87
			CC	267	132				280	119			
			GC/CC	814	413	1.50 (1.23–1.83)	<0.0001	0.03	846	381	1.24 (1.01–1.52)	0.05	0.91
	rs243847	intronic	TT	432	213	[1]			448	197	[1]		
			TC	542	267	0.81 (0.71–0.92)	0.001	0.04	555	254	0.88 (0.76–1.00)	0.05	0.87
			CC	181	66				177	70			
			TC/CC	723	333	0.82 (0.68–0.97)	0.024	0.32	732	324	0.86 (0.71–1.04)	0.11	0.91
*JAG1*	rs6040062	intronic	TT	876	391	[1]			886	381	[1]		
			TG	259	142	1.36 (1.15–1.61)	0.0003	0.02	274	127	1.31 (1.09–1.57)	0.004	0.68
			GG	20	13				20	13			
			TG/GG	279	155	1.38 (1.14–1.67)	0.0007	0.04	294	140	1.29 (1.05–1.59)	0.01	0.68

**Table:** (**b**)

						Overall Survival					Recurrence-Free Survival		
Gene	SNP	Location	Alleles	Alive	Deceased	HR ^1^ (95%-CI)	*p*	*q*	Alive	Deceased	HR ^1^ (95%-CI)	*p*	*q*
*KDR*	rs2305948	V297I	CC	934	424	[1]			944	414	[1]		
			CT	209	113	1.27 (1.06–1.53)	0.01	NA	225	97	1.14 (0.92–1.41)	0.23	NA
			TT	12	9				11	10			
			CT/TT	221	122	1.28 (1.04–1.57)	0.02	NA	236	107	1.09 (0.86–1.37)	0.47	NA

^1^ Hazard ratios for log-additive and dominant models, adjusted for age, sex, stage, grade, BMI, alcohol consumption. Abbreviations: SNP: single nucleotide polymorphism; HR: hazard ration; CI: confidence interval; NA: not applicable.

## Supplementary Materials

Supplementary Materials can be found at https://www.mdpi.com/1422-0067/21/15/5395/s1.

## Abbreviations

CRC	Colorectal cancer
OS	Overall survival
RFS	Recurrence-free survival
SNP	Single nucleotide polymorphism
