# Supplementary material for: Polymorphisms in the Angiogenesis-Related Genes EFNB2, MMP2 and JAG1 Are Associated with Survival of Colorectal Cancer Patients
Source: Int J Mol Sci. 2020 Jul 29;21(15):5395. doi: 10.3390/ijms21155395 (PMC7432124; doi:10.3390/ijms21155395)
Supplement: Supplementary file 1 [file ijms-21-05395-s001.zip › Supplement/EFNB2, MMP2 and JAG1 Supplemenary Figure S1_R1_20200721.pdf]

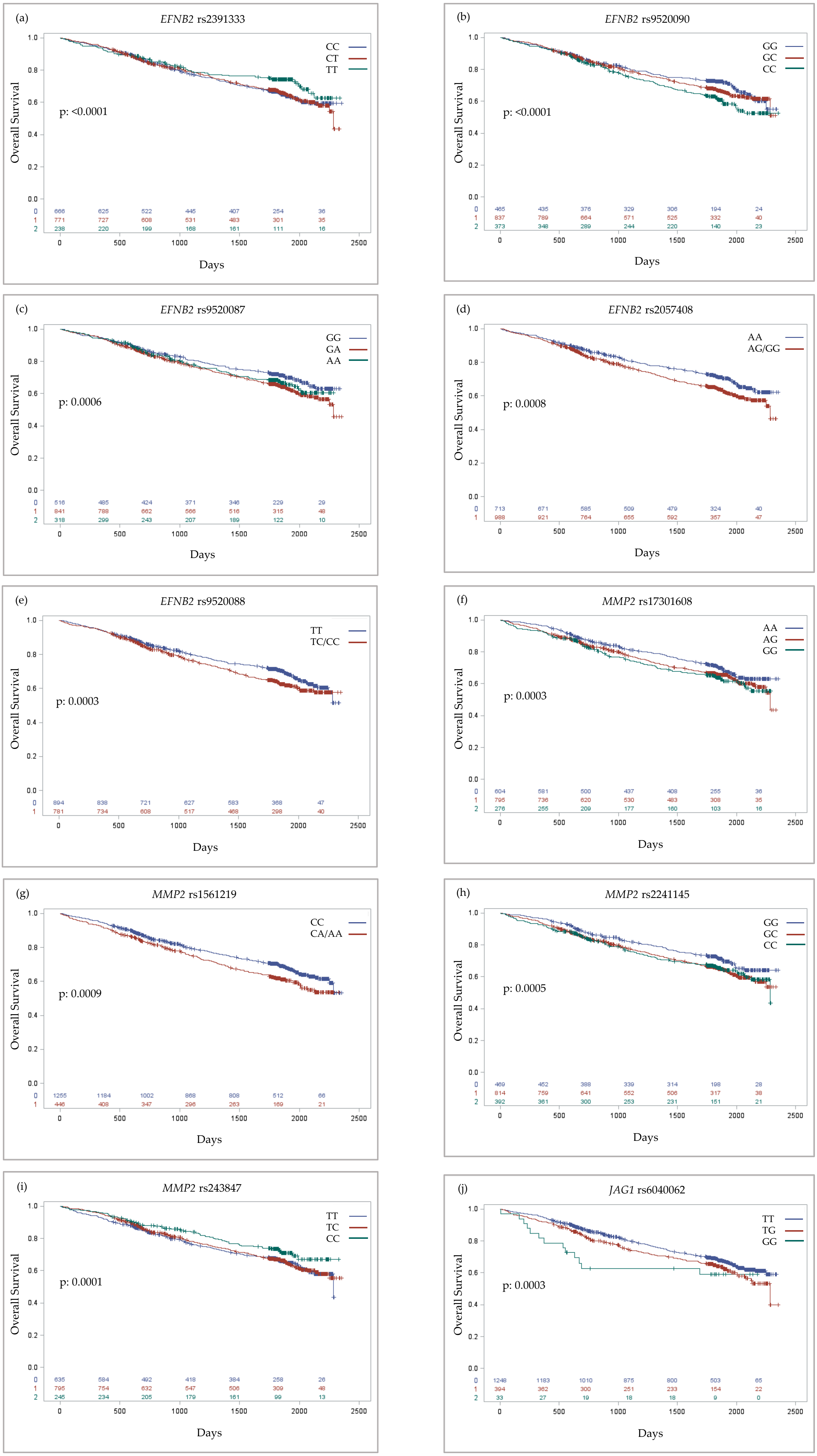


**Supplementary Figure 1**: Kaplan-Meier curves for significant associations with overall survival of colorectal cancer patients. (a) *EFNB2* rs2391333, (b) *EFNB2* rs9520090, (c) *EFNB2* rs9520087, (d) *EFNB2* rs2057408 , (e) *EFNB2* rs9520088, (f) *MMP2* rs17301608, (g) *MMP2* rs1561219, (h) *MMP2* rs2241145, (i) *MMP2* rs243847, and (j) *JAG1* rs6040062
