# Supplementary material for: Polymorphisms in the Angiogenesis-Related Genes EFNB2, MMP2 and JAG1 Are Associated with Survival of Colorectal Cancer Patients
Source: Int J Mol Sci. 2020 Jul 29;21(15):5395. doi: 10.3390/ijms21155395 (PMC7432124; doi:10.3390/ijms21155395)
Supplement: Supplementary file 1 [file ijms-21-05395-s001.zip › Supplement/EFNB2, MMP2 and JAG1 Supplementary Table S1.doc]

**Supplementary Table 1:** Selected genes.

| **Approved Symbol** | **Approved name** | **Chr.** | **NCBI gene ID** | **HGNC ID** |
| --- | --- | --- | --- | --- |
| ***AKT1*** | AKT serine/threonine kinase 1 | 14 | 207 | 391 |
| ***ANGPT1*** | angiopoietin 1 | 8 | 284 | 484 |
| ***ANGPT2*** | angiopoietin 2 | 8 | 285 | 485 |
| ***DKK4*** | dickkopf WNT signaling pathway inhibitor 4 | 8 | 27121 | 2894 |
| ***DLL1*** | delta like canonical Notch ligand 1 | 6 | 28514 | 2908 |
| ***DLL4*** | delta like canonical Notch ligand 4 | 15 | 54567 | 2910 |
| ***EFNB2*** | ephrin B2 | 13 | 1948 | 3227 |
| ***EPHB4*** | EPH receptor B4 | 7 | 2050 | 3395 |
| ***ETS1*** | ETS proto-oncogene 1, transcription factor | 11 | 2113 | 3488 |
| ***FGF2*** | fibroblast growth factor 2 | 4 | 2247 | 3676 |
| ***FLT1*** | fms related tyrosine kinase 1 | 13 | 2321 | 3763 |
| ***FLT4*** | fms related tyrosine kinase 4 | 5 | 2324 | 3767 |
| ***HIF1A*** | hypoxia inducible factor 1 alpha subunit | 14 | 3091 | 4910 |
| ***ID1*** | inhibitor of DNA binding 1, HLH protein | 20 | 3397 | 5360 |
| ***JAG1*** | jagged 1 | 20 | 182 | 6188 |
| ***KDR*** | kinase insert domain receptor | 4 | 3791 | 6307 |
| ***MAPK1*** | mitogen-activated protein kinase 1 | 22 | 5594 | 6871 |
| ***MMP2*** | matrix metallopeptidase 2 | 16 | 4313 | 7166 |
| ***MMP9*** | matrix metallopeptidase 9 | 20 | 4318 | 7176 |
| ***NOTCH4*** | notch 4 | 6 | 4855 | 7884 |
| ***NRP1*** | neuropilin 1 | 10 | 8829 | 8004 |
| ***NRP2*** | neuropilin 2 | 2 | 8828 | 8005 |
| ***PDGFA*** | platelet derived growth factor subunit A | 7 | 5154 | 8799 |
| ***PDGFRB*** | platelet derived growth factor receptor beta | 5 | 5159 | 8804 |
| ***PGF*** | placental growth factor | 14 | 5228 | 8893 |
| ***PIK3CA*** | phosphatidylinositol-4,5-bisphosphate 3-kinase catalytic subunit alpha | 3 | 5290 | 8975 |
| ***PIK3CG*** | phosphatidylinositol-4,5-bisphosphate 3-kinase catalytic subunit gamma | 7 | 5294 | 8978 |
| ***TEK*** | TEK receptor tyrosine kinase | 9 | 7010 | 11724 |
| ***TIE1*** | tyrosine kinase with immunoglobulin like and EGF like domains 1 | 1 | 7075 | 11809 |
| ***VEGFA*** | vascular endothelial growth factor A | 6 | 7422 | 12680 |
| ***VEGFB*** | vascular endothelial growth factor B | 11 | 7423 | 12681 |
| ***VEGFC*** | vascular endothelial growth factor C | 4 | 7424 | 12682 |
| ***VHL*** | von Hippel-Lindau tumor suppressor | 3 | 7428 | 12687 |

Abbreviations: Chr. Chromosome
